# *TLR4/CD14* Variants-Related Serologic and Immunologic Dys-Regulations Predict Severe Sepsis in Febrile De-Compensated Cirrhotic Patients

**DOI:** 10.1371/journal.pone.0166458

**Published:** 2016-11-18

**Authors:** Wen-Chien Fan, Chih-Wei Liu, Shuo-Ming Ou, Chia-Chang Huang, Tzu-Hao Li, Kuei-Chuan Lee, Shiang-Fen Huang, Ying-Ying Yang, Yun-Cheng Hsieh, Shie-Liang Hsieh, Ming-Chih Hou, Han-Chieh Lin

**Affiliations:** 1 Division of Infection, Department of Medicine, Taipei Veterans General Hospital, Taipei, Taiwan; 2 Division of General Medicine, Department of Medicine, Taipei Veterans General Hospital, Taipei, Taiwan; 3 Division of Gastroenterology and Hepatology, Department of Medicine, Taipei Veterans General Hospital, Taipei, Taiwan; 4 Department of Medicine, Taipei Veterans General Hospital, Taipei, Taiwan; 5 Institute of Clinical Medicine, Department of Medicine, National Yang-Ming University School of Medicine, Taipei, Taiwan; 6 Department of Medicine, National Yang-Ming University School of Medicine, Taipei, Taiwan; 7 Genomics Research Center, Academia Sinica, Taipei, Taiwan; Kaohsiung Medical University, TAIWAN

## Abstract

Genetic variants and dysfunctional monocyte had been reported to be associated with infection susceptibility in advanced cirrhotic patients. This study aims to explore genetic predictive markers and relevant immune dysfunction that contributed to severe sepsis in febrile acute de-compensated cirrhotic patents. Polymorphism analysis of candidate genes was undergone in 108 febrile acute de-compensated cirrhotic patients and 121 healthy volunteers. Various plasma inflammatory/regulatory cytokines, proportion of classical (CD 16^-^, phagocytic) and non-classical (CD16^+^, inflammatory) monocytes, lipopolysaccharide (LPS)-stimulated toll-like receptor 4 (TLR4) and intracellular/extracellular cytokines on cultured non-classical monocytes, mCD14/HLA-DR expression and phagocytosis of classical monocytes were measured. For *TLR4*+896A/G variant allele carriers with severe sepsis, high plasma endotoxin/IL-10 inhibits HLA-DR expression and impaired phagocytosis were noted in their classical monocyte. In the same group, increased non-classical monocyte subset, enhanced LPS-stimulated TLR4 expression and TNFα/nitrite production, and systemic inflammation [high plasma soluble CD14 (sCD14) and total nitric oxide (NOx) levels] were noted. For *CD14*-159C/T variant allele carriers with severe sepsis, persist endotoxemia inhibited mCD14/HLA-DR expression and impaired phagocytosis of their classical monocyte. In the same group, increased non-classical monocyte subset up-regulated TLR4-NFκB-iNOS and p38MAPK pathway, stimulated TNFα/nitrite production and elicited systemic inflammation. In febrile acute de-compensated cirrhotic patients, *TLR4*+896A/G and *CD14*-159C/T polymorphisms-related non-classical and classical monocytes dysfunction resulted in increased severe sepsis risk. Malnutrition, high plasma endotoxin and sCD14 levels, single *TLR4*+896A/G or *CD14*-159C/T variant allele carriers and double variant allele carriers are significant predictive factors for the development of severe sepsis among them.

## Introduction

Acute de-compensated cirrhotic patients are suffered from compromise hepatic immune surveillance, impaired phagocytosis and high severe sepsis-related mortality [1]. Poor response to bacterial challenge, increased susceptibility to bacterial infection, high severe sepsis-related mortality had been reported in advanced cirrhotic patients [2,3]. Fever is the nonspecific clinical manifestations for infection that frequently elicits suspicion of sepsis. So, it is important to search accurate predictors to early identify those febrile cirrhotic patients who are at high severe sepsis risk [2,3].

In response to bacterial invasion, Toll-like receptor-4(TLR4)/MD-2 complex and its co-receptor membrane-bound CD14 (mCD14) on leukocyte recognizes lipopolysaccharide (LPS) to activate NFκB-mediated pro-inflammatory signaling and phagocytosis [4–7]. An association between *TLR4* genetic polymorphism and infection susceptibility has been reported in patients with and without cirrhosis [8–10]. Two forms of CD14 had been reported with soluble molecule (sCD14) and mCD14. It had been reported that *CD14*-159C/T polymorphism can affect mCD14 expression, plasma sCD14 levels, increased septic shock susceptibility [11–14].

The circulating non-classical (CD16^+^, inflammatory) monocytes produce pro-inflammatory cytokines TNFα and IL-6 and, *in vitro*, after LPS stimulation. Significantly increased CD16^+^ monocytes proportion can be found in sepsis and cirrhotic patients [15–17]. To initiate effective phagocytosis, HLA-DR expression on monocytes are essential for the presentation of peptides derived from ingested microbes to prevent severe sepsis [1,2]. So, low HLA-DR expression has been reported as poor prognostic markers for ICU mortaility in critically ill cirrhotic patients [18].

Variant allele of *TNF*α -308G/A affects TNFα transcription and increase septic shock susceptibility [19]. Elevated IL1β level enhances the severity of sepsis [20]. IL-6 stimulates TNFα and IL-1β release to amplify the inflammatory reaction. Both *IL-6*-597G/A and *IL-6*-174G/C polymorphism regulated IL-6 transcription rate and plasma IL-6 level, which is elevated in septic patients [21]. High plasma IL-6 helps in detecting severe sepsis in admitted patients with the suspicion of infection [22].

In febrile acute de-compensated cirrhotic patients, current study wants to explore the potential roles of candidate genetic variants and corresponding dys-regulation in the pathogenesis of increased severe sepsis risk. The interactions between genetic variants-related downstream *in vivo* and *in vitro* serologic and immunologic pathogenic changes among cases with and without sever sepsis were assessed.

## Materials and Methods

The detail description was shown in the S1 File.

### Patients and clinical data

Between May 1, 2013 and March 1, 2016, 108 febrile cirrhotic patients, aged between 38 and 80 years, admitted to our hospital for the treatment of an acute de-compensation [ascites, jaundice, hepatic encephalopathy (HE), spontaneous bacterial peritonitis (SBP) variceal bleeding, and hepatorenal syndrome (HRS)] were enrolled consecutively [23]. The diagnosis of cirrhosis was based on liver biopsy results or on clinical (presence of stigma of cirrhosis including spider angioma, plamar erythema, captus medusa, ascites, varcies, splenomegaly, etc), laboratory, and ultrasonographic (including elastrography) data. Fever was defined as body temperature above 39°C or above 38.5°C measured consecutively at two occasions at least 1 h apart. With distribution (30:70) of variant and wild-type alleles frequency of all tested gene polymorphisms, we estimated the number of febrile de-compensated cirrhotic patients needed to observe differences in susceptibility of severe sepsis at least 10% (D) and with a common variance of 20 (σ) by literatures [8,10,12] With the type 1 error risk of 5% (α), power of 80% (1-β) and, Type 2 error (β), 20%; it was estimated that 104 patients would be required in total. Notably, the power (80%) calculation used was sufficient to detect 40% (70–30%) difference in the allele frequency of all tested gene polymorphisms.

Exclusion criteria were: human immunodeficiency virus infection, previous transplantation or any other type of immunodeficiency, steroid treatment, pituitary or adrenal disease, hepatocellular carcinoma, severe chronic heart (New York Heart Association function class III or IV) or pulmonary disease (global initiative for chronic obstructive lung disease III or IV), chronic dialysis, acute respiratory distress syndrome, and refusal of patient to participate. Patients gave written informed consent to participate in the study which was approved by the Institutional Review Board Taipei Veterans General Hospital, Taiwan, R.O.C. (IRB number: 201303013AC, approved on 19/April/2013).

Demographic and the baseline clinical evaluation [the Child-Pugh, and model for end stage liver disease (MELD), APACH III scores] were completed within 48 hours of hospitalization. Subjective global nutritional assessment (SGNA) score of >1 (2 to 4) was defined as malnourish [24]. All clinical parameters especially newly developed systemic inflammatory response syndrome (SIRS) [24], sepsis and severe sepsis during admission and during 3-month follow-up; in-hospital and 3-month mortality and causes of death were carefully collected after enrolled. Sepsis was diagnosed as the presence of SIRS in combination with suspected or proven infection but without any evidence of organ dysfunction or the need for intravenous vasopressor drug support to maintain blood pressure. Severe sepsis was defined as sepsis that was temporally accompanied by the need for intravenous vasopressor drug support (excluding dopamine at ≦5μg/kg/min) to maintain blood pressure (despite adequate fluid resuscitation) along with the presence of perfusion abnormalities, or metabolic acidosis (pH≦7.3) or the development of respiratory, renal, hepatic, or hematological failure. After recruitment, admitted febrile de-compensated cirrhotic patients [25] were divided into severe sepsis group and non-severe sepsis group, which including uncomplicated, SIRS and sepsis cases. Among 108 enrolled febrile de-compensated cirrhotic patients, 9 uncomplicated cases, 18 SIRS cases and 34 sepsis cases were identified as non-severe sepsis group. By contrast, 47 severe sepsis cases were identified as severe sepsis group

Retrospectively, we determined the time of the first de-compensation of cirrhosis (ascites, variceal bleeding, encephalopathy or infection) and the period from this time until the first day of the present hospitalization/time of entering current study (pre-study period). Previous incidence and total episode of infections during the pre-study period were recorded.

Additionally, age and sex-matched unrelated 121 healthy controls and 51 afebrile compensated cirrhotic patients with available blood sample for genetic analysis were included as comparison group. Healthy controls were those whose visit our hospital for health check-up without clinical or biochemical evidence of liver, renal and cardiovascular disease. The afebrile compensated cirrhotic patients were identified from ongoing studies on cirrhosis in outpatient department of our hospital.

### Genetic and serologic analysis

We selected candidate SNPs (S1 Fig) within bacterial recognition [(*TLR4* +896A/G, Asp299Gly substitution mutation, rs4986790; *TLR4* 3′UTR, G/C, rs11536889) &(*CD14*, promoter region,-159C/T, rs2569190; *CD14*, 3′UTR, C/A, rs2563298)] and inflammatory response [(*TNFα* -308G/A, rs1800629; -238G/A, rs361525) & (*IL-1β*, -31, 5′-UTR, T/C, transition, rs1143627; *IL-1β*, +3954C/T, rs1143634) & (*IL-6*, -174G/C transversion, promoter region, rs1800795; *IL-6*, -597G/A, rs1800797)] genes that had available polymorphic information for a Han population by using Applied Biosystems SNP browser software version 3.0 [26].

Plasma soluble CD14 levels, IL-6, IL-1*β*, TNFα, IL-10 and total nitric oxide (NOx, nrtrite+nitrate), and endotoxin were measured by kits purchased from Biosource (USA), R&D Systems (Minneapolis, USA) and Lonza, Walkersville (MD).

### Proportion of CD16^-^ (classical) and CD16^+^ (non-classical) monocyte subsets

Individual′s peripheral blood mononuclear cells (PBMC) were isolated and CD14^+^CD16^−^ and CD14^+^CD16^+^cells were separated by magnetic cell sorting, using MACS isolation kits by negative selection (Miltenyi Biotec, Bergisch Gladbach, Germany) for comparison between groups.

### TLR4/HLA-DR expression on monocytes

The isolated CD16^−^ and CD16^+^ monocytes were double-stained with anti-CD16-PE (BD Biosciences, USA) and anti-TLR4-APC (BD Biosciences, USA) antibodies. In a dose-finding preliminary experiment, the most potent stimulation of TLR4 expression on cells analyzed by flow cytometry (FACScan, BD Biosciences) was presented at 100ng/mL of *Escherichia Coli* LPS (Sigma-Aldrich, St, Louis, MO). Therefore, all the following acute *in vitro* experiments were divided into un-stimulated and stimulated [LPS, 100ng/mL] groups. The HLA-DR expression on CD16^-^ (classical) and CD16^+^ (non-classical) monocytes were measured by staining with anti-HLA-DR-APC, anti-CD14-FITC (BD Biosciences, USA) and anti-CD16-PE antibodies.

Our preliminary experiments revealed that the TLR4 expression was not different between CD16^-^ (classical) monocytes between groups. It is well-established that classical monocyte are phagocytic with no inflammatory attributes [15–17], so the LPS-stimulated extra- and intra-cellular inflammatory cytokines production and corresponding signals were only evaluated in cultured CD16^+^ (non-classical) monocytes.

### Extracellular and intracellular cytokine assays of CD16^+^ monocytes

For both stimulated LPS (100ng/mL) and un-stimulated groups, the isolated CD16^+^ monocytes were incubated for 20 h, cell free supernatants were harvested and analyzed for IL-6, IL-1*β*, and TNFα production using a commercial ELISA kit (R&D, Minneapolis, MN). Additionally, supernatants nitrite concentration was measured by the Griess reaction.

The isolated CD16^***+***^ monocytes from individual subjects were stimulated with LPS (100ng/mL) for 5h. Manensin (1.7μg/mL; Sigma-Aldrich) was added to inhibit cytokine secretion. The CD16^*+*^ cells were stained with anti-TNFα-APC (BD Biosciences, USA) or anti-iNOS-APC **(**LifeSpan Biosciences, USA) with istotype control for 20 min at 4°C, fixed, permeabilized washed, and analyzed by flow cytometry.

### Inflammatory mRNA and protein expressions in cultured CD16^+^monocytes

With and without LPS (100ng/mL) stimulation, *NFkB-p65*, *iNOS*, *p38MAPKα*:, *p38MAPKβ*, *ERK1 mRNA/*protein expressions in isolated CD16^+^ monocytes were measured with primers listed in S1 Table and antibodies purchased from R&D system, Minneapolis, MN; Abcam, Cambridge, MA, UK.

### Membrane-bounded CD14 (mCD14) expression on classical CD16^-^ (phagocytic) monocytes

Both for un-stimulated and stimulated (100ng/mL of LPS) groups of isolated CD16^-^ monocytes, mCD14 expression was analyzed using the phycoerythrin (PE)-labeled anti-CD14 antibody (R&D Systems, Minneapolis, MN, USA) [27,28].

### Phagocytic ability of CD16^-^ (phagocytic) monocytes

Both for un-stimulated and stimulated (100ng/mL of LPS) groups, CD16^-^ monocytes (2×10^5^/well) were incubated in five replicates with Alexa Fluor 488 (AF488)-conjugated *Escherichia coli* BioParticles (Invitrogen). CD16^-^ monocytes incubated with bioiparticles were used as positive controls, while monocytes without bioparticles were used as negative controls. The phagocytic indexes of the CD16^-^ monocytes from different individual were calculated by the following formula, Phagocytic index = [(MFI of experiment-MFI of negative controls)/MFI of positive controls-MFI of negative controls]×100%, where MFI stands for mean fluorescence intensity [29].

### Statistical analysis

The significance of differences in allelic frequencies between each group was determined by Fisher’s exact test. Differences in the distribution of alleles between the groups and deviation from Hardy-Weinberg equilibrium were assessed by Pearson χ2 test and likelihood-ratio χ2 tests of independence; 2×2 tables were used to compare allele distribution between any 2 groups. Continuous variables were expressed as mean and standard deviation (SD). Student′s *t*-test was used to compare continuous variables from two groups. For univariate and multivariate regression analysis, the third quartile of plasma sCD14 (3.7 μg/mL) and endotoxin (>2.3 EU/mL) levels at inclusion of all febrile acute de-compensated cirrhotic patients were used as cut-off values for high-risk group of severe sepsis.

## Results

### Basal Characteristics

Basically, the enrolled afebrile compensated cirrhotic patients were characterized by lower Child-Pugh class, lower MELD score, relative normal serum sodium concentration, higher serum albumin, lower serum bilirubin and less prothrombin time prolongation than febrile de-compensated cirrhotic patients (S2 Table). At inclusion, cases complicated with severe sepsis had significantly higher plasma sCD14, IL-10, NOx levels, higher proportion of moderate/massive ascites, lower serum albumin and more episodes of overall previous infection per patients than those non-severe sepsis cases (S2 Table and S1F Fig). Moreover, the APACH (acute physiologic and chronic health) III score (21.3±7.4 *vs*. 20.6±7.6), the SGNA (subjective global nutritional assessment) score (2.8±0.5 *vs*. 2.3±1.1), percentages of using beta-blockers/diuretics (60/64% *vs*. 65/50%), using oral antibiotics prophylaxis for SBP recurrence at inclusion (24% *vs*. 21%), percentage of previous ascite/variceal bleeding/SBP/encephalopathy (33/80/33/47% *vs*. 40/83/38/56%), causes of current admission [ascites/jaundice/encephalopathy/variceal bleeding/SBP/HRS (25/28/14/27/4/2% *vs*. 22/32/12/24/6/4%)], duration (months) from first de-compensation to entering study, serum bilirubin, prothrombin time prolongation,8plasma CRP level (3.6±0.8 *vs*. 2.9±1.7mg/dL) and plasma TNFα/IL-6/IL-1β levels were not different between febrile de-compensated cirrhotic patients with and without severe sepsis (S2 Table, S1G, S2A and S2C Figs).

### Distribution of variant allele of candidate SNPs between groups

In comparison with healthy controls, higher frequencies of *TLR4*+896A/G and *CD14*-159C/T variant alleles were noted among severe sepsis and non-severe sepsis febrile de-compensated cirrhotic patients as well as afebrile compensated cirrhotic patients (Table 1 and S3 Table). Higher frequencies of *TLR4*+896A/G and *CD14*-159C/T variant alleles were noted in febrile de-compensated cases than afebrile compensated cases (Table 1). In comparison with non-severe sepsis cases, higher frequencies of *TLR4*+896A/G and *CD14*-159C/T variant alleles were noted in severe sepsis cases. However, the percentage of carrying variant alleles of the following SNPs including *TLR4* 3′UTR, G/C; *CD14* ′UTR, C/A; *TNFα* -238G/A; *IL-1β* -31T/C; *IL-1β* +3954C/T; *IL-6* -174G/C; *IL-6* -597G/A were not different among healthy controls, severe sepsis febrile de-compensated cirrhotic patients, non-severe sepsis febrile de-compensated cirrhotic patients and afebrile compensated cirrhotic patients (Table 1 and S3 Table).

**Table 1:** Comparison of distribution (%) of variant allele carriers of candidate SNPs among groups.

	Severe sepsis cases (n = 47)	Non-severe sepsis cases (n = 61)	Significance (severe *vs*. non-severe sepsis cases)	Febrile de-compensated cirrhotic patients (n = 108)	Afebrile compensated cirrhotic patients (n = 51)	Significance (febrile *vs*. afebrile cases)	Healthy controls (n = 121)	Significance (healthy controls *vs*. afebrile cases)
*TLR4* +896A/G	16 (34%) ^##^^,^**	15(25%) ^#^	<0.001	31(29%) ^‡^	10(20%)	0.018	17(14%) ^‡^	0.04
G–allele carriers			3.7 [0.23–4.25]			2.13 [1.302–4.908]		2.05 [1.34–5.17]
*TLR4* 3′UTR, G/C	10(21%)	12(20%)	0.28	22(20%)	8(16%)	0.15	13(11%)	0.08
C–allele carriers			1.89 [0.65–5.96]			1.28 [0.49–3.82]		1.23 [0.57–2.69]
*CD14* -159C/T	18 (38%) ^##^^,^**	15(25%) ^#^	<0.001	33(31%) ^‡^	12(24%)	0.029	21(17%) ^‡^	0.032
T-allele carriers			2.7 [1.1–3.9]			1.98 [1.234–8.214]		1.75 [1.07–4.23]
*CD14* 3′UTR,C/A	8(17%)	9(15%)	0.15	17(16%)	7(14%)	0.36	13(11%)	0.19
A-allele carriers			1.47 [0.94–2.3]			0.89 [0.22–3.36]		1.06 [0.78–1.43]
*TNFα* -308G/A	14 (30%) ^#^	16 (26%)	0.305	30(28%)	14(27%)	0.31	28(23%)	0.166
A-allele carriers			1.01 [099–1.028]			1.16 [1.25–5.13]		1.27 [0.94–1.73]

Cases: de-compensated cirrhotic patients; categorical variables were expressed as case number and percentage [%, case No. of variant allele carrier/variant+wild-type allele carriers] of variant alleles carriers; the unlisted case No. (%) of corresponding wild-type allele carriers were case number in different groups minus variant allele carriers {100-[% of variant allele carriers]}; severe sepsis/non-severe sepsis cases: febrile de-compensated cirrhotic patients with severe sepsis/without severe sepsis

^#^*P* <0.01 &

^##^*P* <0.001 *vs*. healthy controls

*P <0.01 &

**P <0.001 *vs*. non-severe sepsis cases.

‡*P*<0.05 *vs*. afebrile compensated cirrhotic patients; Descriptive significance between groups were showed as *P*-value [odd ratio, OR (95% confidence interval, CI)].

### Characteristics of cases with different genotypes

Significantly, more overall previous episodes of infection (S1F Fig), higher proportion of previous SBP episodes, and severe sepsis cases were observed in *TLR4*+896A/G and *CD14*-159C/T variant allele carriers than wild-type carriers (Table 2). Among *TLR4+896A/G* and *CD14-159C/T* variant allele carriers, significantly higher plasma sCD14 and NOx levels were noted than their comparative groups. Plasma IL-10 level was significantly higher in *TLR4*+896A/G variant allele carrier than their controls but similar between variant and wild-type *CD14*-159C/T allele carriers (Table 2).

**Table 2:** Characteristics of febrile acute de-compensated cirrhotic patients with variant/wild-type allele carriers (variant/wild-type).

Different carrier of *TLR4* +896A/G polymorphism	variant allele (n = 22)	wild-type allele (n = 86)	*P values*
Gender (male/female) ratio	15/7 (68/32%)	62/24 (72/28%)	0.35
Child-Pugh class (A/B+C) (%)	5/17(13/77%)	19/67(22/78%)	0.48
Severity of ascites (no+mild/moderate+massive)(%)	9/13(41/59%)	40/46(47/53%)	0.65
Model of end stage liver disease (MELD) score	17.9± 6.4	19.3± 4.1	0.33
Acute physiologic and chronic health (APACH) III score	22.1 ±5.9	19.8± 6.5	0.24
[C-reactive protein, (CRP)] mg/dL	4.1±1.1	3.9 ±1.7	0.41
[sCD14] μg/mL	6.9±0.5*	3.7±0.4	0.002
[IL-10] pg/mL	17.5 ±2.2*	9.7±1.4	0.01
[total nitric oxide (NOx)] μM	38.5± 3.5*	19.3 ±8.7	0.005
Previous spontaneous bacterial peritonitis (SBP)	10(45%)**	29(34%)	<0.001
Percentage of cases complicated with severe sepsis (%)	13(59%)*	27(31%)	0.005
**Different carrier of *CD14* -159C/T polymorphism**	**variant allele (n = 33)**	**wild-type allele (n = 75)**	
Gender (male/female) ratio	22/11(67/33%)	55/20(73/27%)	0.95
Child-Pugh class (A/B+C) (%)	5/28(15/85%)	19/56(25/75%)	0.69
Ascites (no+mild/moderate+massive)(%)	13/20(39/61%)	36/39(48/52%)	0.38
MELD score	18.3± 2.5	18.4± 5.6	0.52
APACH III score	22.3 ±5.4	24.1±6.3	0.29
[CRP] mg/dL	5.7±1.9	4.6 ±2.1	0.26
[sCD14] μg/mL	4.7±0.8*	1.5±0.4	0.003
[IL-10] pg/mL	16.8 ±1.3	14.3±5.7	0.15
[NOx] μM	37.5 ±2.9*	21.4 ±6.8	0.012
Previous SBP	13(39%)**	21(28%)	<0.001
Percentage of cases complicated with severe sepsis (%)	14(42%)*	23(31%)	0.026

Data are mean±SD; categorical variables were expressed as case number [percentage (%) of frequency]

*P <0.01

**P <0.001 *vs*. *TLR4* +896 A/G&*CD14* -159C/T, wild-type allele carriers.

### Increased CD16^+^ (non-classical) monocyte subset was associated with high TLR4/TNFα/iNOS expression and extracellular TNFα/nitrite production

The baseline and LPS-stimulated TLR4 expression on the CD16^-^ (classical) monocytes were not different between variant and wild-type allele carriers of *TLR4*+896A/G or *CD14*-159C/T as well as between non-severe sepsis and severe sepsis cases (data not shown).

**Fig 1:**
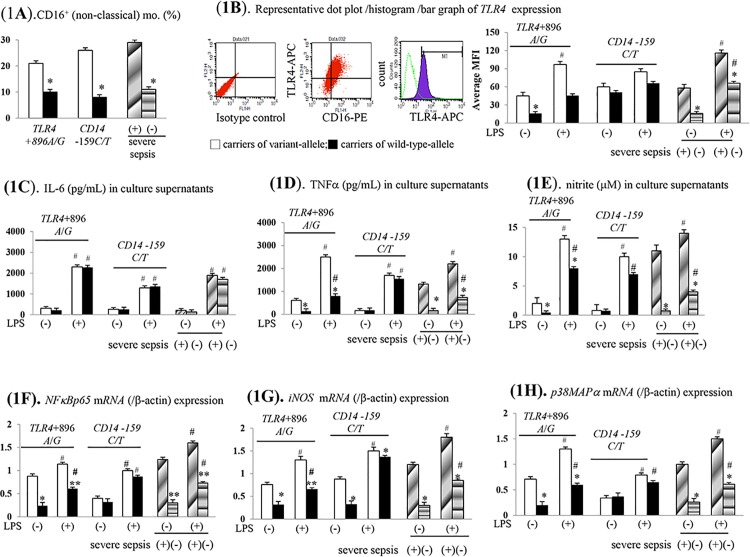
Various LPS-stimulated pro-inflammatory profiles on CD16^+^ (non-classical, inflammatory) monocytes of different cases. (**A**). percentage (%) of CD16^+^ monocyte subset; (**B**). representative flow cytometric dot plots/histograms of TLR4 expression (10000 counts); (**C-E**). LPS-stimulated extracellular production of various substances. (**F-H**). various *mRNA* expressions; *or** *P* < 0.05 or 0.01 *vs*. *TLR4*/*CD14* variant alleles carriers/severe sepsis cases; ^#^ *P* <0.05 *vs*. un-stimulated groups; MFI: mean fluorescence intensity.

**Fig 2:**
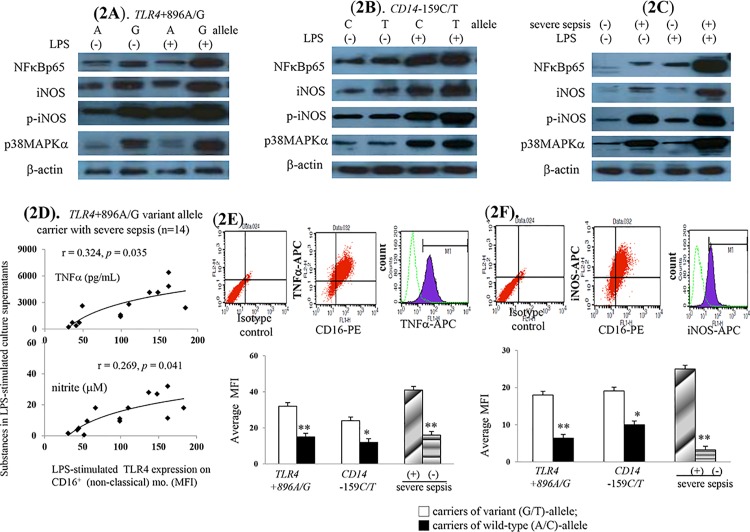
Contribution of increased CD16^+^ (non-classical, inflammatory) monocytes subsets on the increased severe sepsis risk among *TLR4*+896A/G or *CD14*-159C/T variant alleles carriers. (**A-C**). LPS-stimulated various protein expressions on CD16^+^ monocytes of all cases; (**D**). positive correlation between LPS-stimulated TLR4 expression and TNFα/nitrite production; (**E,F**). increased LPS-stimulated intracellular TNFα/iNOS levels. *or** *P* < 0.05 or *P* < 0.001 *vs*. *TLR4*/*CD14* variant alleles carriers/severe sepsis cases.

Significantly, increased proportion of CD16^+^ monocyte subsets were observed in *TLR4*+896A/G or *CD14*-159C/T variant allele carriers and severe sepsis cases (Fig 1A). In supernatant on cultured CD16^+^ monocytes of variant *TLR4*+896A/G allele carriers and severe sepsis cases, significantly higher baseline and LPS-stimulated TLR4 over-expression was accompanied by TNFα and nitrite over-production (Fig 1B & 1D & 1E). Corresponding to the enhanced LPS-stimulated TNFα/nitrite production, the significantly up-regulated NFκBp65, p-iNOS and p38MAPα *mRNA* and protein expressions were found on cultured monocyte of *TLR4*+896A/G variant allele carriers and severe sepsis cases (Fig 1F–1H, Fig 2A–2C).

In variant *TLR4*+896 allele carriers with severe sepsis, a significantly positive correlation was noted between LPS-stimulated TLR4 expression and corresponding TNFα/nitrite production (Fig 2D). However, above correlation was loss among variant *CD14*-159C/T allele carriers with severe sepsis (S3B Fig). In line with increased extracellular LPS-stimulated TNFα/nitrite levels, significantly higher intracellular TNFα/iNOS level confirm the cellular sources of TNFα/nitrite from circulating CD16^+^ monocytes of these cases (Fig 2E & 2F).

Compared to un-stimulated states, higher LPS-stimulated IL-6/IL-1β levels in CD16^+^ monocytes supernatant was observed among all cases. Nonetheless, the magnitudes of these LPS-stimulated changes were not different between variant and wild-type *TLR4*+896A/G or *CD14*-159C/T allele carriers as well as between non-severe sepsis and severe sepsis cases (Fig 1C, S2D Fig).

Significantly, acute LPS incubation stimulated the TNFα, iNOS, TNFα/nitrite, *NFκBp65*, *iNOS and p38MAPα* in CD16^+^ monocytes of both variant and wild-type *CD14*-159C/T allele carriers (Fig 1B & 1D–1H). Nonetheless, the baseline and LPS-stimulated above mention signals were not different between variant and wild-type *CD14*-159C/T allele carriers (Fig 1B & 1D–1H).

### Down-regulated HLA-DR expression on monocytes increase severe sepsis risk

**Fig 3:**
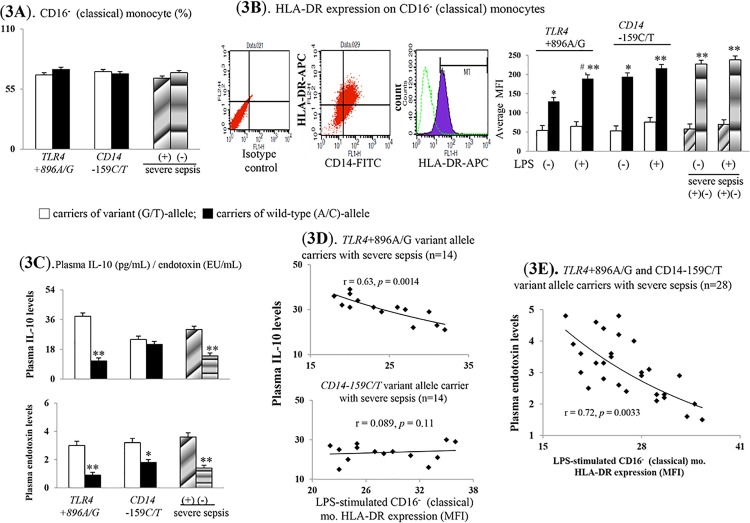
Profound pro-inflammatory responses are accompanied by CD16^-^ classical (phagocytic) monocyte-related abnormalities. (**A**). percentage (%) of CD16^-^ monocyte subset; (**B**). surface HLA-DR expression on CD16^-^ monocyte; (**C**). plasma endotoxin and IL-10 levels; correlation between LPS-stimulated surface HLA-DR expression on CD16^-^ monocyte and plasma IL-10 (**D**)/endotoxin (**E**) levels. *or** *P* < 0.05 or *P* < 0.001 *vs*. *TLR4*/*CD14* variant alleles carriers/severe sepsis cases. ^#^ *P* <0.05 *vs*. un-stimulated groups.

Fig 3A observed that the CD16^-^ monocyte subset proportions were not different between cases carrying *TLR4*+896A/G or *CD14*-159C/T variant allele and severe sepsis. Both on the CD16^-^ and CD16^+^ monocyte, the significantly lower baseline and LPS-stimulated HLA-DR expression were observed in cases carrying variant *TLR4*+896A/G or *CD14*-159C/T allele carriers and severe sepsis (Fig 3B, S3A Fig).

Significantly, higher plasma endotoxin and IL-10 levels were observed in cases carrying variant *TLR4*+896A/G allele and severe sepsis (Fig 3C). Among variant *CD14*-159C/T allele carriers, higher plasma endotoxin levels rather than plasma IL-10 levels were noted than wild-type carriers.

Among *TLR4*+896A/G variant allele carriers with severe sepsis, a significant negative correlation was noted between plasma IL-10 levels and LPS-stimulated HLA-DR expression on CD16^-^ monocyte (Fig 3D). Among both *TLR4*+896A/G and *CD14*-159C/T variant allele carriers with severe sepsis, a significant negative correlation was found between plasma endotoxin level and LPS-stimulated HLA-DR expression on CD16^-^ monocyte (Fig 3E).

### Decreased mCD14 density on CD16^-^ (classical) monocyte

**Fig 4:**
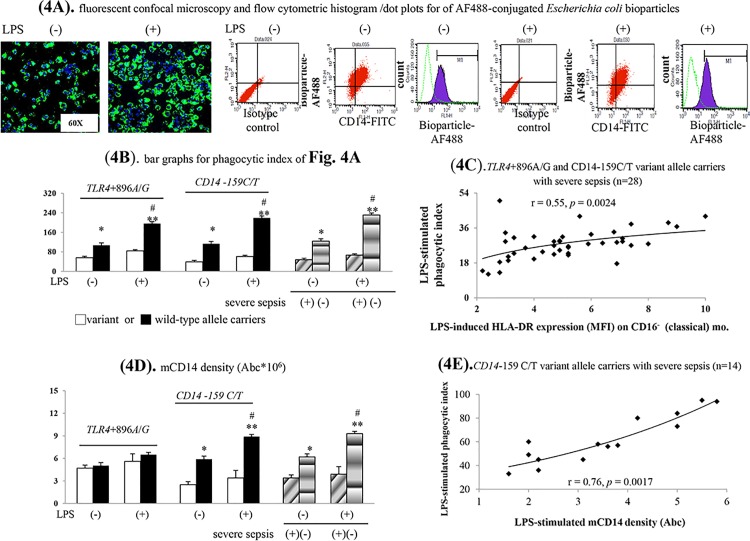
Various phagocytic profiles of CD16^-^ (classical, phagocytic) monocyte of different cases. (**A**).representative photomicrographs/flow cytometric histogram/dot plots and (**B**) bar graph for phagocytic index; correlation between LPS-stimulated surface HLA-DR expression on CD16^-^ monocyte (**C**)/mCD14 density (**E)** and phagocytic index; (**D**). LPS-stimulated membrane bound-CD14 densities. Abc: antigen binding capacity; * *P* < 0.05 or ** *P* < 0.001 *vs*. *TLR4*/*CD14* genes variant alleles carriers/severe sepsis cases; ^#^ *P* <0.05 *vs*. un-stimulated groups.

Among cultured CD16^-^ monocytes collected from cases carrying wild-type *TLR4*+896A/G or *CD14*-159C/T allele and non-severe sepsis, acute LPS incubation significantly stimulated HLA-DR/mCD14 expressions and phagocytosis (Figs 3B, 4B & 4D). Nonetheless, above mention phenomena was disappeared among CD16^-^ monocytes collected from cases carrying variant *TLR4*+896A/G or *CD14*-159C/T allele and severe sepsis.

The phagocytic abilities of CD16^-^ monocyte were significantly decreased among cases carrying variant *TLR4*+896A/G or *CD14*-159C/T allele and severe sepsis (Fig 4A & 4B). Notably, the magnitude of LPS-stimulated HLA-DR expression determined the phagocytosis of CD16^-^ monocyte in *TLR4*+896A/G and *CD14*-159C/T variant allele carriers with severe sepsis (Fig 4C).

Additionally, the mCD14 density was remarkably decreased on CD16^-^ monocyte of *CD14*-159C/T variant allele carriers and severe sepsis cases (Fig 4D). Further analysis indicated that there was a positive correlation between LPS-stimulated mCD14 densities and phagocytic index on CD16^-^ monocyte in *CD14*-159C/T variant allele carriers with severe sepsis (Fig 4E). By contrast, there were no correlation between LPS-stimulated mCD14 densities and phagocytic index on CD16^-^ monocyte in *TLR4*+896A/G variant allele carriers with severe sepsis (S3C Fig).

#### Summative mechanisms for severe sepsis risk in cases (Fig 5)

Both *TLR4*+896A/G and *CD14*-159C/T variant allele carriers shared common increased CD16^+^ (non-classical, inflammatory) monocyte subset–related mechanisms for increased severe sepsis risk (Figs 1B, 2D–2F). The up-regulated TLR4 expression-increased TNFα/iNOS/nitrite levels and subsequently systemic acute inflammatory status (high plasma sCD14 and total NO levels) resulted in severe sepsis among them (Figs 1 & 2, S2 Table, Table 2). High plasma endotoxin/IL-10-related HLA-DR down-regulation and impaired phagocytosis of CD16^-^ (classical, phagocytic) monocytes also resulted in increased severe sepsis risk among *TLR4*+896A/G and *CD14*-159C/T variant allele carriers (Figs 3, 4A & 4B). Further, low mCD14 density on CD16^-^ (phagocytic) monocytes contributed to increased severe sepsis risk in *CD14*-159C/T variant allele carriers (Figs 3 & 4).

**Fig 5:**
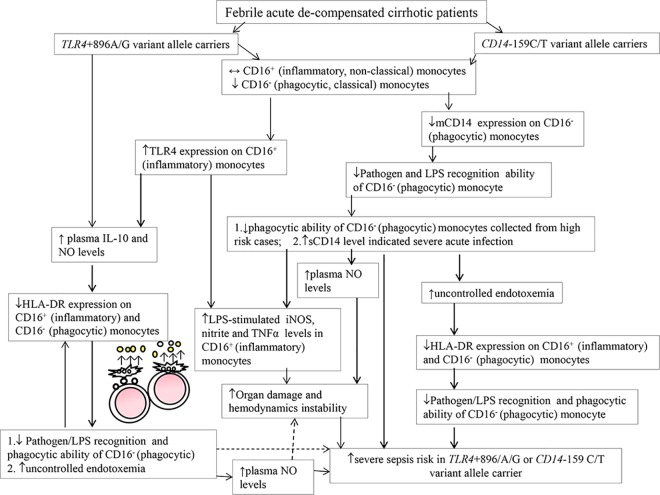
Schematic representation of key different findings compared to corresponding wild-type allele carriers for the mechanisms of increase severe sepsis risk in febrile acute de-compensated cirrhotic patients carrying *TLR4*+896/A/G or *CD14*-159C/T variant alleles in current study. *TLR4*: gene of toll-like receptor 4; LPS: lipopolysaccharide.

### Predictors for severe sepsis

Among all cases, 59% of *TLR4*+896A/G variant allele carriers complicated with severe sepsis whereas 43% of *CD14*-159C/T variant allele carriers complicated with severe sepsis (Table 2).

In univariate analysis, the odd ratio for predicting severe sepsis among *TLR4*+896A/G variant allele carriers was 2.41 whereas 1.82 for *CD14*-159C/T variant allele carriers (Table 3). Additionally, 13% of them were double *TLR4*+896A/G and *CD14*-159C/T variant allele carriers. Accordingly, higher percentage (69%) of double *TLR*+896A/G and *CD14*-159C/T variant allele carriers were complicated with severe sepsis compared to either single *TLR*+896A/G or *CD14*-159C/T variant alleles carriers (59% or43%). In multivariate analysis, malnutrition (SGNA score>1), high plasma sCD14 level (>3.7 μg/mL), high endotoxin level (>2.3 EU/mL), single or double *TLR4* +896A/G or *CD14* -159C/T variant alleles carriers were associated with increased severe sepsis risk in febrile de-compensated cirrhotic patients (Table 3).

**Table 3:** Regression analysis of predictive factors for complicated with severe sepsis in febrile de-compensated cirrhotic patients.

Parameter at inclusion	Odd Ratio(95% CI)		*P values*
**(A). Univariate analysis**			
Child-Pugh class B+C		1.23(0.41–2.16)	0.81
Moderate+massive ascites		1.18 (0.82–1.75)	0.53
Model of end stage liver disease (MELD) score		0.943(0.36–1.89)	0.25
Plasma acute C-reactive protein level (CRP, mg/dL)		0.67(0.94–4.67)	0.79
Acute physiologic and chronic health (APACH) III score		1.58(1.04–2.24)	0.23
High [sCD14] (>3.7μg/mL)		1.73 (0.42–2.13)	0.007
High [endotoxin] (>2.3 EU/mL)		1.36 (1.08–2.3)	0.014
Subjective global nutritional assessment (SGNA) score>1		2.688(0.244–2.87)	0.029
Previous spontaneous bacterial peritonitis (SBP)		0.842(1.18–2.34)	0.45
*TLR4* +896 A/G, variant(G)–allele carriers		2.41 (0.275–2.71)	0.02
*CD14*–159 C/T, variant ((T)-allele carriers		1.82(0.783–2.51)	0.01
Double *TLR4* +896A/G and *CD14* -159C/T variant–alleles carriers		4.37 (0.25–1.27)	0.0039
**(B). Multivariate analysis**			
Subjective global nutritional assessment (SGNA) score>1		3.714(0.737–4.123)	0.0014
High [sCD14] (>3.7μg/mL)		2.198(0.045–2.65)	0.006
High [endotoxin] (>2.3 EU/mL)		3.105(0.89–3.142)	0.0032
*TLR4* +896 A/G, variant ((G)–allele carriers		2.967(0.593–5.123)	0.004
*CD14*–159 C/T, variant ((T)-allele carriers		3.11(0.71–5.2)	0.001
Double *TLR4* +896A/G and *CD14* -159C/T variant–alleles carriers		5.44(0.065–3.1)	0.002

SGNA score of >1 (2 to 4) indicated malnutrition; cut-off values for high plasma IL-10 and endotoxin levels were defined as greater than third quartile of data of severe sepsis and non-severe sepsis case.

## Discussion

Early innate immune responses to LPS are critical for determining resistance to bacterial infection; the same responses are important driving forces behind the pathophysiology of sepsis in infected individuals. Unregulated responses to bacterial agonists can cause immune-suppression, which may lead to secondary infections, or to over-stimulated inflammatory responses that can cause severe sepsis, shock, and death in cirrhosis and non-cirrhosis [5–7,30–32].

Guarner-Argente *et al*. reported a trend towards a higher incidence of bacterial infections in de-compensated cirrhotic patients whose carrying TLR4 D299G variant genotype compared to wild-type carriers [10]. Appenrodt *et al*. has demonstrated an association between NOD2 (nucleotide-binding oligomerization domain containing 2) variants and the SBP-related mortality in de-compensated cirrhotic patients [33]. However, the roles and immunologic mechanisms of genetic polymorphism on the increased severe sepsis risk had never been explored in febrile acute de-compensated cirrhotic patients.

Thoughtfully, the correlation among the plasma sCD14/endotoxin/NOx levels and various immune regulatory effectors (LPS-stimulated *TLR4*, mCD14, *NFκBp65* and *iNOS* expression and corresponding intracellular and extracellular IL-6, TNFα, nitrite levels on cultured classical and non-classical monocytes) activities were surveyed in our current study. Notably, current study revealed that variant alleles of *TLR4*+896A/G and *CD14*-159C/T modulate immunologic protein abundance (increase plasma sCD14 levels/up-regulated TLR4 expression on non-classical inflammatory monocyte and down-regulated HLA-DR and mCD14 expression on classical monocyte) and function (impaired phagocytosis of classical monocytes) in our febrile acute de-compensated cirrhotic patients with severe sepsis.

It was suggested that chronic endotoxemia is associated with increased plasma IL-10 levels in cirrhotic patients [30,34]. In de-compensated cirrhotic patients, high IL-10 levels had been reported to be associated with decreased monocyte phagocytic ability [1,9,30]. In line with previous studies, carriers of variant allele of *TLR4* +896A/G in our febrile acute de-compensated cirrhotic patients did show increased LPS-stimulated IL-10 production and complicated with higher frequency (59%) of severe sepsis compared to wild-type allele carriers (32%).

For host protection from pathogen, the LPS-stimulated NFκB/MAP kinases and TNF-α release depend on the up-regulation of TLR4 expression on activated monocytes [4,5,9]. Sequentially, the up-regulated NFκB-MAP kinases, over-produced TNF-α and iNOS-derived NO (nitrite) involved in the development of severe sepsis and septic shock [35–38]. Not surprisingly, in our *TLR4*+896A/G and *CD14*-159C/T variant alleles carriers with severe sepsis, increased *TLR4* expression was accompanied by the up-regulation of *in vitro* LPS-stimulated NFκB, TNF-α, and iNOS signals on their cultured non-classical (CD16^+^, inflammatory) monocytes.

Membrane bound CD14 (mCD14) are pattern recognition receptors generate early innate immune response against bacterial pathogens. Decreased mCD14 expression on activated classical (phagocytic) monocyte may decrease the pathogen elimination ability of individuals. Previous study had revealed elevated levels of sCD14 in patients with sepsis [39]. Additionally, low monocyte mCD14 and high plasma sCD14 level can predict 28-day mortality in patients with community acquired infections [27,28]. Notably, in our febrile acute de-compensated cirrhotic patients with severe sepsis, low mD14 expression is characterized by the decreased LPS-stimulated phagocytic ability of classical monocyte, whereas high plasma sCD14 level indicated uncontrolled sepsis among *CD14*-159C/T variant allele carriers.

Reduced monocyte HLA-DR expression has been reported in patients with septic de-compensation of acute-on-chronic liver failure [30]. In advanced cirrhotic patients, high plasma IL-10 levels can negatively regulated their HLA-DR expression on monocytes [30]. In our *TLR4*+896A/G variant allele carriers with severe sepsis, a negative correlation was noted between plasma IL-10 and LPS-stimulated CD16^-^ (classical) monocyte HLA-DR expression. Due to persistent endotoxemia, acute de-compensated cirrhotic patients were suffered from the down-regulation of monocyte HLA-DR expression and immune paralysis [1,30].

Accordingly, it was reasonable to observe a significant negative correlation between plasma endotoxin levels and LPS-stimulated CD16^-^ (classical) monocyte HLA-DR expression in our *TLR4*+896A/G or *CD14*-159C/T variant allele carriers with severe sepsis. It had been reported that iNOS-derived NO can down-regulate HLA-DR expression and suppress systemic monocyte activation [40]. In our *TLR4*+896A/G or *CD14*-159C/T variant allele carriers with severe sepsis, the significantly higher LPS-stimulated iNOS expression and nitrite production on their cultured non-classical (inflammatory) monocytes and significantly higher plasma NOx levels compared to their control groups were observed.

Our study revealed that LPS-stimulated IL-1β/IL-6 production in cultured supernatant of non-classical monocyte and plasma IL-1β/IL-6 levels were not significantly different between our *TLR4*+896A/G or *CD14*-159C/T variant and wild-type allele carriers as well as between severe sepsis and non-severe sepsis cases. Actually, recent meta-analysis reported a lack of association between *IL-6* -174G/C polymorphism and sepsis risk [41]. In our study, the lack of association between the *TNFα*-308G/A, *TNFα*-238G/A, *IL-6*-174G/C, *IL-6*-597G/A, *IL-1β*-31T/C and +3954C/T polymorphisms and severe sepsis risk suggested that these inflammatory cytokines might influence sepsis progression via mechanisms other than regulations by these polymorphisms. Probably, the phenotypic heterogeneity of the sepsis syndrome including the infection location, and the amount of time passed since the onset of infection, as well as other individual parameters including background and environmental factors, contributed to some dis-concordance with previous reports.

Serum CRP level, MELD score and APACH score, were well known as a predictor of severity of inflammation/disease. However, the parameters revealed non-significance to predict severe sepsis in current study. In fact, the study about the predictor for severe sepsis in febrile de-compensated cirrhotic patients is limited. It had been suggested that the concentration of lipopolysaccharide binding protein (LBP) is associated inversely with disease severity scores and outcomes in critically ill cirrhotic patients with severe sepsis [42]. Another study reported that serum LPB predicts severe bacterial infection in cirrhotic patients with ascites [43]. In non-cirrhotic patients, serum C-reactive protein (CRP) level had been reported as the risk factor for severe sepsis in children with cancer and febrile neutropenia [44]. Nonetheless, CRP is primarily produced in the liver and its specificity as diagnostic and prognostic tool for infection is limited in cirrhotic patients with compromised liver function, which will underestimate the severity of acute infection. MELD score is a representative maker for cirrhosis severity as well as criteria in donor liver allocation systems. In liver transplant recipients, MELD score >23 is reported to predict ICU stay >10 days without modify survival [45]. The APACHE is ICU-specific prognostic scores. In cirrhotic patients admitted to ICU, MELD score predict short mortality better than APACH scores [46]. In our admitted febrile de-compensated cirrhotic patients, the lack of roles of serum CRP level, MELD score and APACH score in prediction svere sepsis might be due to different studied population contrast to other non-cirrhotic critically ill patients.

Using multi-modalities comprehensive approaches, our data showed the contribution on the *TLR4*+896A/G and *CD14*-159C/T polymorphism-related immune dysfunction including increased non-classical (inflammatory) monocyte proportion-related LPS hyper-inflammatory response and decreased classical (phagocytic) monocyte proportion-related impaired phagocytosis in febrile acute de-compensated cirrhotic patients complicated with severe sepsis. In addition to regular predictive factors such as malnutrition, high plasma endotoxin and sCD14 levels for development of severe sepsis in high risk groups, the novel predictive factors were either single or double *TLR4*+896A/G and *CD14*-159C/T variant alleles carriers observed in current study. Accordingly, treatment strategies should change from uniform management to rapid stratifications and sub-categorization, with subsequent aggressive targeted therapeutic intervention in those most at risk.

## Supporting Information

S1 Fig **(A-E) Schematic representative SNPs that explored within various cytokines genes in current study**; the comparison of (**F**) previous episode of infection during the *pre-study period* and (**G**) duration of first de-compensation to entering study (*pre-study period*) between cases with and without severe sepsis. * *P* < 0.05 or ** *P* < 0.001 *vs*. *TLR4*/*CD14* variant alleles carriers/severe sepsis cases. *Pre-study period*: first de-compensation of cirrhosis and the period from this time until the first day of the present hospitalization/time of entering current study.
(TIF)

S2 Fig **(A-C).** Plasma TNF*α*, IL-6 and IL-1β levels of all cases; (**D**). LPS-stimulated IL-1β production; (**E,F**). LPS-stimulated p38MAPβ and ERK1 *mRNA* expression on CD16^+^ (non-classical) monocytes of all cases; ^#^*p*<0.05 *vs*. un-stimulated group.
(TIF)

S3 Fig (**A**). surface HLA-DR expression on CD16+ monocyte; (**B**).correlation between LPS-stimulated surface HLA-DR expression on CD16^-^ monocyte and levels of TNF*α*/nitrite in the LPS-stimulated on the culture supernatant in *CD14*-159C/T variant allele carrier with severe sepsis. (C). correlation between LPS-stimulated mCD14^+^ density (Abc) on CD16^-^ (classical) monocyte and LPS-stimulated phagocytic index *TLR4*+896A/G variant allele carriers with severe sepsis. * *P* < 0.05 *vs*. *TLR4*/*CD14* variant alleles carriers/severe sepsis cases; ^#^*p*<0.05 *vs*. un-stimulated group.
(TIF)

S1 Table Specific primers used in this study. (DOCX)

S2 Table Characteristics of all cases (cirrhotic patients) and healthy controls at inclusion. (DOCX)

S3 Table Comparison of distribution (%) of variant allele carriers of candidate SNPs among groups. (DOCX)

S1 File Supplementary Materials and Methods. (DOCX)
