# Supplementary figures and images for: TLR4/CD14 Variants-Related Serologic and Immunologic Dys-Regulations Predict Severe Sepsis in Febrile De-Compensated Cirrhotic Patients
Source: PLoS One. 2016 Nov 18;11(11):e0166458. doi: 10.1371/journal.pone.0166458 (PMC5115743; doi:10.1371/journal.pone.0166458)

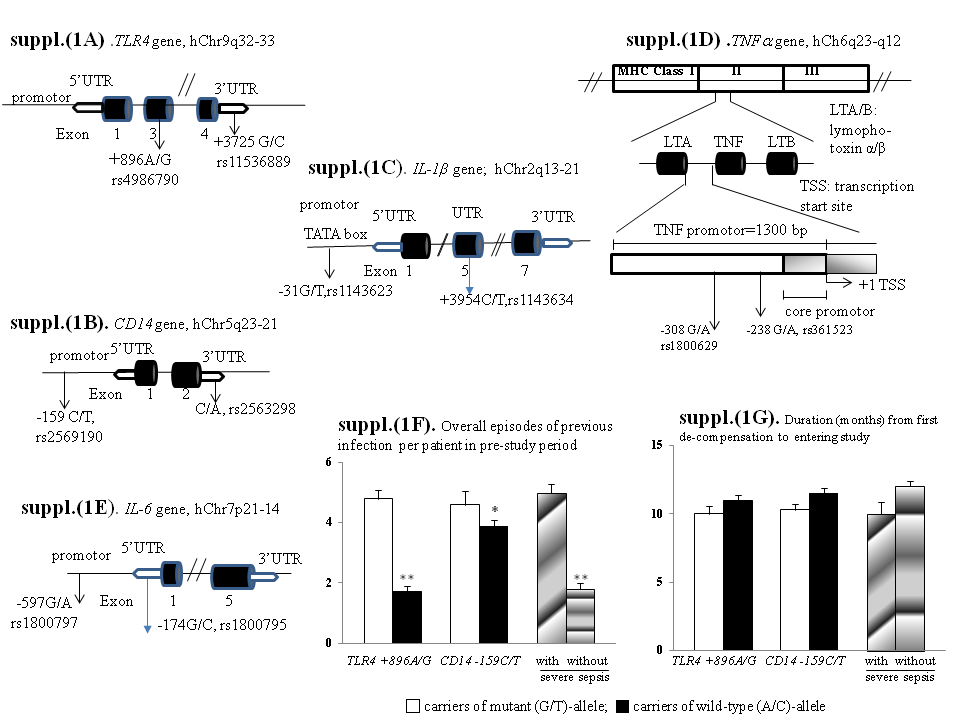

Supplement: S1 Fig — (A-E) Schematic representative SNPs that explored within various cytokines genes in current study; the comparison of (F) previous episode of infection during the pre-study period and (G) duration of first de-compensation to entering study (pre-study period) between cases with and without severe sepsis. * P < 0.05 or ** P < 0.001 vs. TLR4/CD14 variant alleles carriers/severe sepsis cases. Pre-study period: first de-compensation of cirrhosis and the period from this time until the first day of the present hospitalization/time of entering current study. (TIF) [file pone.0166458.s001.TIF]

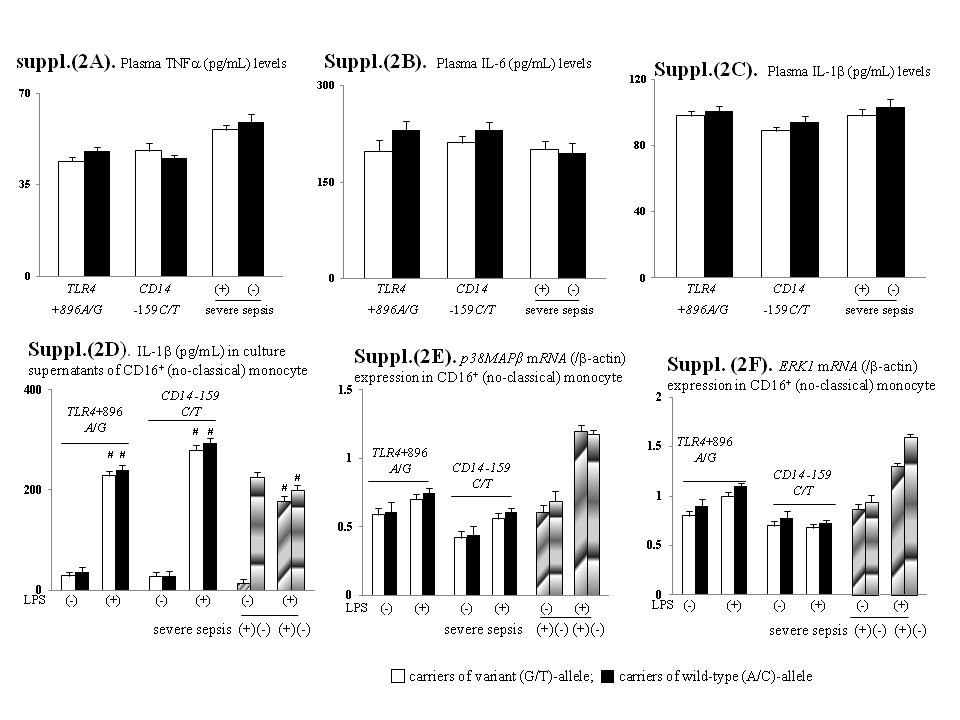

Supplement: S2 Fig — (A-C). Plasma TNFα, IL-6 and IL-1β levels of all cases; (D). LPS-stimulated IL-1β production; (E,F). LPS-stimulated p38MAPβ and ERK1 mRNA expression on CD16+ (non-classical) monocytes of all cases; #p<0.05 vs. un-stimulated group. (TIF) [file pone.0166458.s002.TIF]

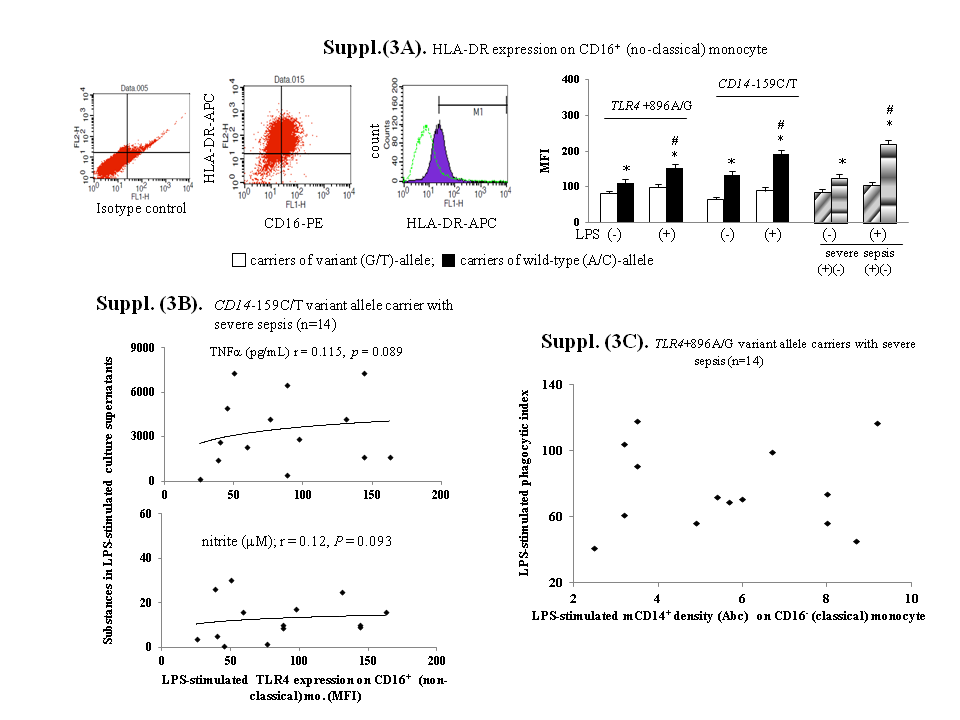

Supplement: S3 Fig — (A). surface HLA-DR expression on CD16+ monocyte; (B).correlation between LPS-stimulated surface HLA-DR expression on CD16- monocyte and levels of TNFα/nitrite in the LPS-stimulated on the culture supernatant in CD14-159C/T variant allele carrier with severe sepsis. (C). correlation between LPS-stimulated mCD14+ density (Abc) on CD16- (classical) monocyte and LPS-stimulated phagocytic index TLR4+896A/G variant allele carriers with severe sepsis. * P < 0.05 vs. TLR4/CD14 variant alleles carriers/severe sepsis cases; #p<0.05 vs. un-stimulated group. (TIF) [file pone.0166458.s003.TIF]
