# Supplementary material for: TLR4/CD14 Variants-Related Serologic and Immunologic Dys-Regulations Predict Severe Sepsis in Febrile De-Compensated Cirrhotic Patients
Source: PLoS One. 2016 Nov 18;11(11):e0166458. doi: 10.1371/journal.pone.0166458 (PMC5115743; doi:10.1371/journal.pone.0166458)
Supplement: S1 Table — (DOCX) [file pone.0166458.s004.docx]

**S1 Table.** Specific primers used in this study

| ***Gene names*** | **Sequences of Sense** | **Sequences of antisense** |
| --- | --- | --- |
| *NFkB-p65* | 5′-CACCTCAATGGCTACACAGGACCA-3′ | 5′-ATCTTGAGCTCGGCAGTGTT-3′ |
| *iNOS* | 5′ GCTGGACGATGAAGATTTCC-3′ | 5′ ATTGTCAGACAGGTCTAGGC-3′′ |
| *p38MAPKα* | 5′-ACAAGACTGCCTGGAGCTA-3′ | 5′-CCAATGACGTTCTCATGGTG-3′ |
| *38MAPKβ* | 5′-CGTGTACCTGGTGACCCTCT-3′ | 5′-AGCCACGTAGCCTGTCATCT-3′ |
| *ERK1* | 5′-TCCAAGGGCTACACCAAATC-3′ | 5′-TCCAAGGGCTACACCAAATC-3′ |
| *β-actin* | 5′-AGATCAAGATCATTGCTCCTCCTG-3′ | 5′-CATTTGCGGTGGACGATGGA-3′ |
