# Supplementary material for: TLR4/CD14 Variants-Related Serologic and Immunologic Dys-Regulations Predict Severe Sepsis in Febrile De-Compensated Cirrhotic Patients
Source: PLoS One. 2016 Nov 18;11(11):e0166458. doi: 10.1371/journal.pone.0166458 (PMC5115743; doi:10.1371/journal.pone.0166458)
Supplement: S2 Table — (DOCX) [file pone.0166458.s005.docx]

**S2 Table.** Characteristics of all cases (cirrhotic patients) and healthy controls at inclusion

|  | **Severe sepsis cases ( n=47)** | **Non- severe sepsis cases (n=61)** | **Febrile de-compensated cases (n=108)** | **Afebrile compensated cases (n=51)** | **Healthy controls (n=121)** |
| --- | --- | --- | --- | --- | --- |
| Age (year) | 60.1±11.5 | 58.5± 9.5 | 59.2 ±8.7 | 56.9±10 | 51.7±13 |
| Gender (male/female) ratio | 33/14(70/30%) | 44/17(72/28%) | 77/31(71/29%) | 35/16(69/31%) | 65/56(54/46%) |
| Child-Pugh class (A/B+C) (%) | 10/37(21/79%) | 14/47(23/77%) | 24/84(22/78%)‡ | 44/7(86/14%) | - |
| Severity of ascites (no+mild/moderate+massive) | 14/33(30/70%)* | 35/26(57/43%) | 49/59(45/55%)‡ | 51/0(100/0%) | - |
| MELD score | 17.5± 6.7 | 18.5± 4.7 | 18.1± 3.6‡ | 10.6±2.9 | - |
| [sodium] (mEq/L) | 131.5± 5.7 | 132.3 ±6.3 | 131.9 ±4.7‡ | 138.4±1.5 | 139±4 |
| [BUN] (mg/dL) | 12.3 ±4.9 | 11.6 ±5.3 | 11.9±8.2 | 14.6± 0.9 | 14.2±3.6 |
| [Creatinine] (mg/dL) | 1.62 ±0.84 | 1.32±1.01 | 1.45±0.96 | 1.2±0.4 | 0.72±0.31 |
| White blood cell count (/cumm) | 10600±565 | 9800±872 | 10048±352 | 3982±574 | 4859±306 |
| [albumin] (g/dL) | 2.7±0.2* | 3.1±0.6 | 2.9±0.3‡ | 3.8±0.5 | 4.6±0.4 |
| [bilirubin] (mg/dL) | 3.8±1.9 | 3.2±0.8 | 3.5±1.1‡ | 2.6±0.9 | 0.7±0.5 |
| Prothrombin time prolonged (sec.) | 3.5±0.7 | 3.1±0.5 | 3.6±0.8‡ | 1.8±0.2 | - |
| [sCD14] level (μg/mL) | 6.1±0.3* | 1.5±0.6 | 3.5 ± 0.8 | - | - |
| [IL-10] (pg/mL) | 18.1 ±1.6* | 10.4± 2.3 | 13.6± 4.1 | - | - |
| [NOx] (μM) | 43.1±7.9* | 16.2± 4.8 | 27.9 ±6.3 | - | - |

Data are mean±SD; Categorical variables were expressed as case number [percentage (%) of frequency];severe sepsis/non-severe sepsis cases: febrile de-compensated cirrhotic patients with severe sepsis/without severe sepsis; MELD: model of end stage liver disease; BUN: blood urea nitrogen; NOx: total nitric oxide; **P* <0.01 *vs*. non-severe sepsis cases; ‡*P*<0.05 *vs.* afebrile compensated cirrhotic patients; afebrile compensated cirrhotic patients and healthy controls whose have available blood sample for genetic analysis were retrospectively obtained from medical records for a period of 10 days in the period of collecting blood samples for genetic analysis. The retrospective data with less than 5% of missing data were included.
