# Supplementary material for: TLR4/CD14 Variants-Related Serologic and Immunologic Dys-Regulations Predict Severe Sepsis in Febrile De-Compensated Cirrhotic Patients
Source: PLoS One. 2016 Nov 18;11(11):e0166458. doi: 10.1371/journal.pone.0166458 (PMC5115743; doi:10.1371/journal.pone.0166458)
Supplement: S3 Table — (DOCX) [file pone.0166458.s006.docx]

**S3 Table.** Comparison of distribution (%) of variant allele carriers of candidate SNPs among groups

|  | **severe**  **sepsis**  **cases**  **(n=47)** | **Non-severe**  **Sepsis**  **Cases**  **(n=61)** | **Significance**  **(severe**  ***vs*. non-severe**  **sepsis cases)** | **Febrile de-compensated cases (n=108)** | **Afebrile compensated cases (n=51)** | **Significance (febrile de-compensate**  ***vs*. afebrile compensated cases)** | **Healthy**  **controls**  **(n= 121)** | **Significance (healthy controls**  ***vs*. afebrile compensated cases)** |
| --- | --- | --- | --- | --- | --- | --- | --- | --- |
| *TNFα* -238G/A | 11(23%) | 21 (34%) | 0.339 | 32(30%) | 14(27%) | 0.181 | 25(21%) | 0.03 |
| A-allele carriers |  |  | 1.197  [ 0.828-1.729] |  |  | 1.04  [0.56-1.95] |  | 1.98  [1.13-3.46] |
| *IL-1β*,  -31T/C, | 19 (40%) | 24 (39%) | 0.68 | 43(40%) | 19(37%) | 0.318 | 45(37%) | 0.637 |
| C -allele  carriers |  |  | 0.92[0.8-2.1] |  |  | 1.07  [0.96-1.19] |  | 0.96  [0.86-1.37] |
| *IL-1β*,  +3954C/T | 23 (49%) | 24 (39%) | 0.28 | 47(44%) | 21(41%) | 0.427 | 54(45%) | 0.322 |
| T –allele  carriers |  |  | 1.3[0.8-2.4] |  |  | 1.16  [0.95-1.4] |  | 1.11  [0.81-1.51] |
| *IL-6*,  -174G/C | 14 (30%) | 20 (33%) | 0.623 | 34(31%) | 17(33%) | 0.333 | 42(35%) | 0.085 |
| C -allele  carriers |  |  | 1.53[0.91-2.57] |  |  | 1.05  [0.89-1.25] |  | 0.78  [0.66-0.99] |
| *IL-6*,  -597G/A | 18 (38%) | 22 (36%) | 0.43 | 40(37%) | 18(35%) | 0.402 | 39 (32%) | 0.513 |
| A-allele  carriers |  |  | 118[0.78-1.77] |  |  | 0.92  [0.88-1.51] |  | 1.49  [0.97-2.29] |

^#^*P* <0.01 & ^##^*P* <0.001 *vs*. healthy controls;**P* <0.01 & ***P* <0.001 *vs*. non-severe sepsis cases. ‡*P*<0.05 *vs.* afebrile compensated cases; Descriptive significance between groups were showed as *P*-value [odd ratio, OR (95% confidence interval, CI)].
