# Supplementary material for: TLR4/CD14 Variants-Related Serologic and Immunologic Dys-Regulations Predict Severe Sepsis in Febrile De-Compensated Cirrhotic Patients
Source: PLoS One. 2016 Nov 18;11(11):e0166458. doi: 10.1371/journal.pone.0166458 (PMC5115743; doi:10.1371/journal.pone.0166458)
Supplement: S1 File — (DOCX) [file pone.0166458.s007.docx]

# S1 file. Supplementary Materials and Methods

## Patients and clinical data

108 febrile cirrhotic patients admitted to our hospital for the treatment of an acute de-compensation [ascites, hepatic encephalopathy (HE), bacterial infection, variceal bleeding, and hepatorenal syndrome (HRS)] were enrolled consecutively. A subgroup of these patients was included in a previous report exploring the roles of serum pentraxin-3 and TNF-like weak inducer of apoptosis (TWEAK) on the prediction of the prognosis of admitted cirrhotic patients with acute de-compensation [1]. Fever was defined as body temperature above 39°C or above 38.5°C measured consecutively at two occasions at least 1 h apart.

Demographic and all clinical parameters from admission, discharged and 3-months follow-up were carefully collected after enrolled the case into this study. Newly developed systemic inflammatory response syndrome (SIRS), sepsis and severe sepsis during admission and during 3-month follow-up; hospital and 3-month mortality and causes of death were identified through medical record.

SIRS, sepsis and severe sepsis were defined according to the American College of Chest Physicians/Society of Critical Care Medicine consensus definitions [2]. SIRS was defined as 2 or more of the following variables: Fever of more than 38°C or less than 36°C; heart rate of more than 90 beats per minute; respiratory rate of more than 20 breaths per minute or arterial carbon dioxide tension (PaCO_2_) of less than 32 mm Hg; abnormal white blood cell count (>12,000/µL or < 4,000/µL or >10% immature [band] forms).

Inclusion and the baseline clinical evaluation were performed within 48 hours of hospitalization and included basic demographic data (age, sex), history and physical examination, liver and renal tests, serum various inflammatory mediators and hemoglobin, ascitic fluid analysis and culture (if available), fresh urine sediment, chest x-ray, and abdominal ultrasonography for primary site of infection. The median of three measured values of mean arterial pressure, heart and respiratory rates and body temperature were recorded to estimate SIRS, sepsis, severe sepsis and APACH III scores [3]. Severity of cirrhosis and liver failure was estimated by the Child-Pugh and the model for end stage liver disease (MELD) scores [4].

Additionally, we retrospectively determined the time of the first de-compensation of cirrhosis (ascites, variceal bleeding, encephalopathy or infection) and the period from this time until the first day of the present hospitalization/time of entering current study (pre-study period). Previous incidence and total number of infections during the pre-study period were recorded by patient interview, careful revision of medical records and the electronic database.

## Genetic analysis

Blood samples were taken from all enrolled cases and controls in this study. Genomic DNA for genotyping was extracted with a DNeasy Blood kit (Qiagen, Valencia, CA). We selected candidate SNPs within bacterial recognition [(*TLR4* +896A/G, Asp299Gly substitution mutation, rs4986790; *TLR4* 3′UTR, G/C, rs11536889) &(*CD14*, promoter region,-159C/T, rs2569190; *CD14*, 3′UTR, C/A, rs2563298)] and inflammatory response [(*TNFα* -308G/A, rs1800629; -238G/A, rs361525) & (*IL-1β*, -31, 5′-UTR, T/C, transition, rs1143627; *IL-1β*, +3954C/T, rs1143634) & (*IL-6*, -174G/C transversion, promoter region, rs1800795; *IL-6*, -597G/A, rs1800797)] genes that had available polymorphic information for a Han population by using Applied Biosystems SNP browser software version 3.0. HapMap genotypes were analyzed in Haploview (Haploview software (<http://www.broad.mit.edu/mpg/haploview/> provided in the public domain by The Broad Institute, Massachusetts Institute of Technology, Cambridge, MA) [6]. SNPs were selected by applying the criteria of a threshold minor allele frequency (MAF) in the HapMap CHB+JPT population of 0.10. The wild-type and mutant alleles of all the above mention SNPs were determined based on available public information and were assessed by the TaqMan Allelic discrimination assay with a commercial kit (ABI), according to the manufacturer′s instruction.

## Inflammatory cytokines

### Plasma soluble CD14 levels, IL-6, IL-1β, TNFα, IL-10 and total nitric oxide (NOx, nrtrite+nitrate) were measured by ELISA kits (Biosource, USA; R&D Systems (Minneapolis, MN). NO levels in plasma are short lived and were thus evaluated by its stable metabolites nitrate (NO_3_) and nitrite (NO_2_) [total NO, (NO_3_+NO_2_)] using the Griess reaction with commercial avaiable colorimetric ELISA kit (Sigma-Aldrich, St. Louis, MO, USA). Additionally, plasma endotoxin concentration was measured by a commercially available kit (Cambrex Limulus Amebocyte Lysate [LAL] kit; Lonza, Walkersville, MD). This assay has a sensitivity range of 0.1–1.0 endotoxin unit (EU)/ml. Normal values from lean subjects measured in our laboratory ranged from 0.15 to 0.35 EU/ml. Inter- and intra-assay variations for this test were <10%.

## Proportion of CD16^-^ (classical)) and CD16^+^ (non-classical) monocyte subsets

Peripheral blood mononuclear cells (PBMC) were isolated from buffy coats of febrile acute de-compensated cirrhotic patients by density-gradient centrifugation of heparinized blood using endotoxin-free Ficoll-Hypaque (GE-Healthcare) according to manufactures protocol. Subsequently, CD56^−^CD16^−^CD14^+^ and CD56^−^CD16^+^ CD14^+^cells [referred to as CD16^-^ (classical)) and CD16^+^ (non-classical) monocytes] were separated by magnetic cell sorting, using MACS isolation kits by negative selection (Miltenyi Biotec, Bergisch Gladbach, Germany). Briefly, PBMC were first incubated with MACS anti-CD56 antibody conjugated to paramagnetic microbeads in order to eliminate the NK (CD16^+^) cell fraction. NK-depleted PBMC were further incubated with MACS anti-CD16 PE-conjugated antibody to isolate CD16^+^ monocytes. CD56^−^CD16^−^ PBMCs were finally incubated with MACS anti-CD14 FITC-conjugated antibody to obtain the CD16^−^CD14^+^ monocyte fraction. Then, the percentages of CD16^-^ and CD16^+^ monocytes between groups were calculated.

## TLR4 and HLA-DR expression on CD16^+^ (non-classical) or CD16^-^ (classical) monocytes

Isolated CD16^+^ and CD 16^-^ monocytes were plated at a density of 1×10^5^ cells per well in a 96-well dish and stimulated with vehicle (0) or 10, 100, 1000 ng/mL ultrapure *Escherichia Coli* LPS (Sigma-Aldrich, St, Louis, MO) in a dose-finding preliminary experiments (triplicate in each group). After incubation for 20 h, the cells were double-stained with anti-CD16-PE (BD Biosciences, USA) and anti-TLR4-APC (BD Biosciences, USA), or isotype-matched IgG monocloned antibodies. After washing in 1% fetal bovine serum (FBS) (Moregte Biotech, Bulimba, Australia) in phosphate-buffered saline (PBS), the cell were re-suspended in 2% formaldehyde for 30 min and washed again. The cells were re-suspended in the solution consisting of 10% dimethyl sulfoxide (DMSO) (Sigma) and 90% FBS, and stored at -80°C for batch analysis. On the day of analysis, all cells were thawed and washed in 1% FBS, and TLR4 expression on the surface of cells were analyzed by flow cytometry (FACScan, BD Biosciences). For flow cytometry the MFI of the isotype control was subtracted from the MFI of antibody-stained cells for each replicate culture. The MFI was compared between groups. Data were analyzed using FlowJo software (Tree Star, Ashland, OR).

## Extracellular cytokine assays of CD16^+^ monocytes

For both stimulated LPS (100ng/mL) and un-stimulated groups, the isolated CD16^+^ monocytes from individual subjects were re-suspended in 10% FBS RPMI 1640 medium (Invitrogen) for 20h and plated at a density of 1×10^5^ cells per well in a 96-well dish. After incubation for 20 h, cell free supernatants were harvested and analyzed for IL-6, IL-1β, and TNFα production using a commercial ELISA kit (R&D, Minneapolis, MN). Additionally, supernatants nitrite concentration was measured by the Griess reaction. Briefly, samples were centrifuged at 1,500 rpm to pellet cells. One hundred microliters of the remaining culture supernatant was mixed with 100 μl of a 1:1 mixture of reagent A (0.2% naphthylethylenediamine dihydrochloride in 5% phosphoric acid) and reagent B (2% sulfanilamide in 5% phosphoric acid). Absorbance at 550 nm was immediately recorded and compared to the absorbance of a freshly prepared standard curve of sodium nitrite.

## Assessment of various mRNA and protein expressions in cultured CD16^+^ (non-classical) monocytes

For individual subjects, total *RNA* and protein were extracted from isolated CD16^+^ monocytes with RNeasy Mini kit (Qiagen) with on-column DNase treatment according to manufacturer′s instruction after stimulation with LPS (100ng/mL). *NFkB-p65, iNOS, p38MAPKα*:*, p38MAPKβ, ERK1 mRNA* expressions were measured with SYBR green rt-qPCR as previously described using β–actin as endogenous control. Specific primers were commercially available from Qiagen (S1 Table). Additionally, various proteins [NFBp65, iNOS, phosphorylated (p)-iNOS, p38MPAKα] were measured with appropriate antibodies purchased from R&D system, Minneapolis, MN; Abcam, Cambridge, MA, UK.

## Membrane-bounded CD14 (mCD14) expression on classical CD16^-^ (phagocytic) monocytes

FACS analysis was performed with an FACS caliber using the CellQuest software (Becton Dickinson, Heidelberg). Both for un-stimulated and stimulated (100ng/mL of LPS) groups of isolated CD16^-^ monocytes, CD14 expression was analyzed using the phycoerythrin (PE)-labeled anti-CD14 antibody (R&D Systems, Minneapolis, MN, USA). The instrument was calibrated using the QuantiBRITE^TM^ PE beads according to the manufacturer′s instructions (Becton Dickinson, Heidelberg). The quantiBRITE^TM^ tube contains a lyophilized pellet of beads conjugated with four standard levels PE: 1 700, 14 000, 39 000 and 133 000 molecules per bead. These beads and the samples were measured with the same instrument settings in the PE fluorescence channel. The beads were used to calculate the number of antigen molecules per cell, expressed as antibody binding capacity (abc), which is, the median number of PE molecules bound by the monocyte [7,8].

## Assessment of the phagocytic ability of classical CD16^-^ (phagocytic) monocytes

Both for un-stimulated and stimulated (100ng/mL of LPS) groups, CD16^-^ monocytes were re-suspended in complete media and cultured in flat-bottom 96-well ELISA plates at a concentration of 2×10^5^ cells/well for about 3-hour. After the cells had adhered, the medium was aspirated. Next, 100μL of pre-cooled FITC-labeled *E coli* bioparticles was mixed with 100μL medium at 37°C for 30 minutes to provide the monocytes with better conditions for engulfment. These Alexa Fluor 488 (AF488)-conjugated *Escherichia coli* BioParticles (Invitrogen) at a ratio of 10:1 in medium were then added to the monocytes that adhered to the wells and phagocytosis was allowed to proceed for 2-hour. Subsequently, excess bioparticles were removed by aspiration. Trypan blue was then added as a quencher and incubated for 2-minutes at room temperature to ensure that extracellular particles were sufficiently quenched. The trypan blue was them removed by aspiration and the fluorescence intensity of the phagocytosed bioparticles was quantitatively measured for 1-sec. using the fluorescence plate reader at 480nm excitation and 520nm emission. Bioparticles in medium only were used as the background level for the tests. CD16^-^ monocytes incubated with bioiparticles were used as positive controls, while monocytes without bioparticles were used as negative controls. All experiments were carried out five replicates.

For fluorescent confocal microscopy-based phagocytosis assay, the detached and paraformaldhyde-fixed monocytes were stained with Hoechst 33342 (blue, nuclear staining) by incubation in PBS. Next, for the flow cytometry dot plots-based phagocytosis assay, the above PBMCs were detached from the 96-well ELISA plates by washing with ice-cold bovine serum albumin-Hanks balanced salt solution, and the detached monocytes were transferred to fluorescence-activated cell sorter (FACS) tubes, fixed with paraformaldhyde (3%, W/V) in PBS, and analyzed by flow cytometry (FACScan; Bacton Dickinson). The phagocytic indexes of the CD16^-^ monocytes from different individual were calculated by the following formula, Phagocytic index = [(MFI of experiment-MFI of negative controls)/MFI of positive controls-MFI of negative controls]×100%, where MFI stands for mean fluorescence intensity [9].

## Statistical analysis

Allele frequencies were obtained by direct count. The significance of differences in allelic frequencies between each group was determined by Fisher’s exact test. Differences in the distribution of alleles between the groups and deviation from Hardy-Weinberg equilibrium were assessed by Pearson χ2 test and likelihood-ratio χ2 tests of independence; 2×2 tables were used to compare allele distribution between any 2 groups. Continuous variables were expressed as mean and standard deviation (SD). Student′s *t*-test was used to compare continuous variables from two groups. All significant tests were two-tailed and were considered statistically significant at *P* <0.05. For univariate and multivariate regression analysis, the third quartile of plasma sCD14 (3.7 μg/mL) and endotoxin (>2.3 EU/mL) levels at inclusion of all febrile acute de-compensated cirrhotic patients were used as cut-off values for high-risk group of severe sepsis.

# References

1. [Fan WC](http://www.ncbi.nlm.nih.gov/pubmed/?term=Fan%20WC%5BAuthor%5D&cauthor=true&cauthor_uid=26872435), [Huang CC](http://www.ncbi.nlm.nih.gov/pubmed/?term=Huang%20CC%5BAuthor%5D&cauthor=true&cauthor_uid=26872435), [Yang YY](http://www.ncbi.nlm.nih.gov/pubmed/?term=Yang%20YY%5BAuthor%5D&cauthor=true&cauthor_uid=26872435), [Lin A](http://www.ncbi.nlm.nih.gov/pubmed/?term=Lin%20A%5BAuthor%5D&cauthor=true&cauthor_uid=26872435), [Lee KC](http://www.ncbi.nlm.nih.gov/pubmed/?term=Lee%20KC%5BAuthor%5D&cauthor=true&cauthor_uid=26872435), [Hsieh YC](http://www.ncbi.nlm.nih.gov/pubmed/?term=Hsieh%20YC%5BAuthor%5D&cauthor=true&cauthor_uid=26872435), et al. Serum pentraxin-3 and tumor necrosis factor-like weak inducer of apoptosis (TWEAK) predict severity of infections in acute decompensated cirrhotic patients. [J Microbiol Immunol Infect](http://www.ncbi.nlm.nih.gov/pubmed/26872435) 2016 (In press) [doi:10.1016/j.jmii.2015.12.006](http://dx.doi.org/10.1016/j.jmii.2015.12.006).
2. American College of Chest Physicians/Society of Critical Care Medicine Consensus Conference. (1992) Definitions for sepsis and organ failure and guidelines for the use of innovative therapies in sepsis. Cri. Care Med 20: 864-874.
3. [Knaus WA](http://www.ncbi.nlm.nih.gov/pubmed/?term=Knaus%20WA%5BAuthor%5D&cauthor=true&cauthor_uid=1959406), [Wagner DP](http://www.ncbi.nlm.nih.gov/pubmed/?term=Wagner%20DP%5BAuthor%5D&cauthor=true&cauthor_uid=1959406), [Draper EA](http://www.ncbi.nlm.nih.gov/pubmed/?term=Draper%20EA%5BAuthor%5D&cauthor=true&cauthor_uid=1959406), [Zimmerman JE](http://www.ncbi.nlm.nih.gov/pubmed/?term=Zimmerman%20JE%5BAuthor%5D&cauthor=true&cauthor_uid=1959406), [Bergner M](http://www.ncbi.nlm.nih.gov/pubmed/?term=Bergner%20M%5BAuthor%5D&cauthor=true&cauthor_uid=1959406), [Bastos PG](http://www.ncbi.nlm.nih.gov/pubmed/?term=Bastos%20PG%5BAuthor%5D&cauthor=true&cauthor_uid=1959406), et al. (1991) The APACHE III prognostic system. Risk prediction of hospital mortality for critically ill hospitalized adults. Chest 100 (6): 1619-38.
4. Moreau R, Jalan R, Gines P, [Pavesi M](http://www.ncbi.nlm.nih.gov/pubmed/?term=Pavesi%20M%5BAuthor%5D&cauthor=true&cauthor_uid=23474284), [Angeli P](http://www.ncbi.nlm.nih.gov/pubmed/?term=Angeli%20P%5BAuthor%5D&cauthor=true&cauthor_uid=23474284), [Cordoba J](http://www.ncbi.nlm.nih.gov/pubmed/?term=Cordoba%20J%5BAuthor%5D&cauthor=true&cauthor_uid=23474284), et al. (2013) Acute-on-chronic liver failure is a distinct syndrome that develops in patients with acute de-compensation of cirrhosis. Gastroenterology 144: 1426–1437.
5. [Gatta A](http://www.ncbi.nlm.nih.gov/pubmed/?term=Gatta%20A%5BAuthor%5D&cauthor=true&cauthor_uid=11399401), [Amodio P](http://www.ncbi.nlm.nih.gov/pubmed/?term=Amodio%20P%5BAuthor%5D&cauthor=true&cauthor_uid=11399401), Merkel C, [Merkel C](http://www.ncbi.nlm.nih.gov/pubmed/?term=Merkel%20C%5BAuthor%5D&cauthor=true&cauthor_uid=11399401), [Di Pascoli L](http://www.ncbi.nlm.nih.gov/pubmed/?term=Di%20Pascoli%20L%5BAuthor%5D&cauthor=true&cauthor_uid=11399401), [Boffo G](http://www.ncbi.nlm.nih.gov/pubmed/?term=Boffo%20G%5BAuthor%5D&cauthor=true&cauthor_uid=11399401), et al. (2001) Nutrition and survival in patients with liver cirrhosis. Nutrition 17(6): 445–450.
6. International HapMap Consortium (2003) The international Hap-Mao project. Nature 426**:** 789-796.
7. [Repo H](http://www.ncbi.nlm.nih.gov/pubmed/?term=Repo%20H%5BAuthor%5D&cauthor=true&cauthor_uid=16283111). (2005) Peripheral blood phagocyte CD14 and CD11b expression on admission to hospital in relation to mortality among patients with community-acquired infection. [Inflamm Res](http://www.ncbi.nlm.nih.gov/pubmed/16283111) 54(10): 428-434.
8. [Aalto H](http://www.ncbi.nlm.nih.gov/pubmed/?term=Aalto%20H%5BAuthor%5D&cauthor=true&cauthor_uid=17577824), [Takala A](http://www.ncbi.nlm.nih.gov/pubmed/?term=Takala%20A%5BAuthor%5D&cauthor=true&cauthor_uid=17577824), [Kautiainen H](http://www.ncbi.nlm.nih.gov/pubmed/?term=Kautiainen%20H%5BAuthor%5D&cauthor=true&cauthor_uid=17577824), [Siitonen S](http://www.ncbi.nlm.nih.gov/pubmed/?term=Siitonen%20S%5BAuthor%5D&cauthor=true&cauthor_uid=17577824), [Repo H](http://www.ncbi.nlm.nih.gov/pubmed/?term=Repo%20H%5BAuthor%5D&cauthor=true&cauthor_uid=17577824). (2007) Monocyte CD14 and soluble CD14 in predicting mortality of patients with severe community acquired infection. [Scand. J Infect Dis](http://www.ncbi.nlm.nih.gov/pubmed/17577824) 39: 596-603.
9. Yang YY, Hsieh SL, Lee PC, [Yeh YC](http://www.ncbi.nlm.nih.gov/pubmed/?term=Yeh%20YC%5BAuthor%5D&cauthor=true&cauthor_uid=24953022), [Lee KC](http://www.ncbi.nlm.nih.gov/pubmed/?term=Lee%20KC%5BAuthor%5D&cauthor=true&cauthor_uid=24953022), [Hsieh YC](http://www.ncbi.nlm.nih.gov/pubmed/?term=Hsieh%20YC%5BAuthor%5D&cauthor=true&cauthor_uid=24953022), et al. (2014) Long-term cannabinoid type 2 receptor agonist therapy decreases bacterial translocation in rats with cirrhosis and ascites. J Hepatol 61(5): 1004-1013.
